# Divergent drivers of the spatial and temporal variations of cropland carbon transfer in Liaoning province, China

**DOI:** 10.1038/s41598-017-13358-4

**Published:** 2017-10-12

**Authors:** Xian-Jin Zhu, Han-Qi Zhang, Tian-Hong Zhao, Jian-Dong Li, Hong Yin

**Affiliations:** College of Agronomy, Shenyang Agricultural University, Shenyang, Liaoning province 110855 China

## Abstract

Spatial and temporal variations are important points of focus in ecological research. Analysing their differences improves our understanding on the variations of ecological phenomena. Using data from the Liaoning Statistical Yearbook, we investigated the spatial and temporal variations of cropland carbon transfer (CCT), an important ecological phenomenon in quantifying the regional carbon budget, in particular, the influencing factors and difference. The results showed that, from 1992 to 2014, the average CCT in Liaoning province was 18.56 TgC yr^−1^ and decreased from northwest to southeast. CCT spatial variation was primarily affected by the ratio of planting area to regional area (RPR) via its effect on the magnitude of carbon transfer (MCT), which depended mainly on fertilizer usage per area (FUA). From 1992 to 2014, CCT exhibited a significantly increasing trend with a rate of 0.48 TgC yr^−1^. The inter-annual variation of CCT was dominated by carbon transfer per planting area (CTP) through its effect on MCT, which significantly correlated with FUA but showed no significant correlation with climatic factors. Therefore, the factors affecting the spatial variation of CCT differed from those that affected its inter-annual variation, indicating that the spatial and temporal variations of ecological phenomena were affected by divergent factors.

## Introduction

Spatial and temporal variations have been a vital focus of ecological research and have attracted much attention^1,2^. Many studies have assumed that the drivers of spatial and temporal variations in ecology are similar and have investigated the temporal variation of ecological phenomena using spatial sampling^3,4^. However, recent studies have showed that the spatial variation of annual gross primary productivity (GPP) is strongly affected by annual mean air temperature (MAT) and annual mean precipitation (MAP)^5,6^, while MAT and MAP contribute little to the inter-annual variation of annual GPP in most ecosystems^7,8^. This highlights the need to illustrate whether drivers of spatial variation differ from those of temporal variation.

Cropland carbon transfer (CCT) is an important component of cropland carbon budget^2,9^ and is composed of the quantity of organic matter harvested from croplands^10,11^ including grains and straws but excluding residues. Investigating the spatiotemporal variations of CCT helps to accurately assess the cropland carbon budget, which is vital in regional carbon budget assessments aiming to mitigate climate change^2,9^. This is because cropland plays a key role in maintaining the global carbon budget^12,13^. In addition, CCT serves as a vital process of global carbon cycle, which is a key topic of ecological research. Analysing the spatiotemporal variations of CCT can also reveal whether there are differences in the drivers of spatial and temporal variations of ecological phenomena.

**Figure 1:**
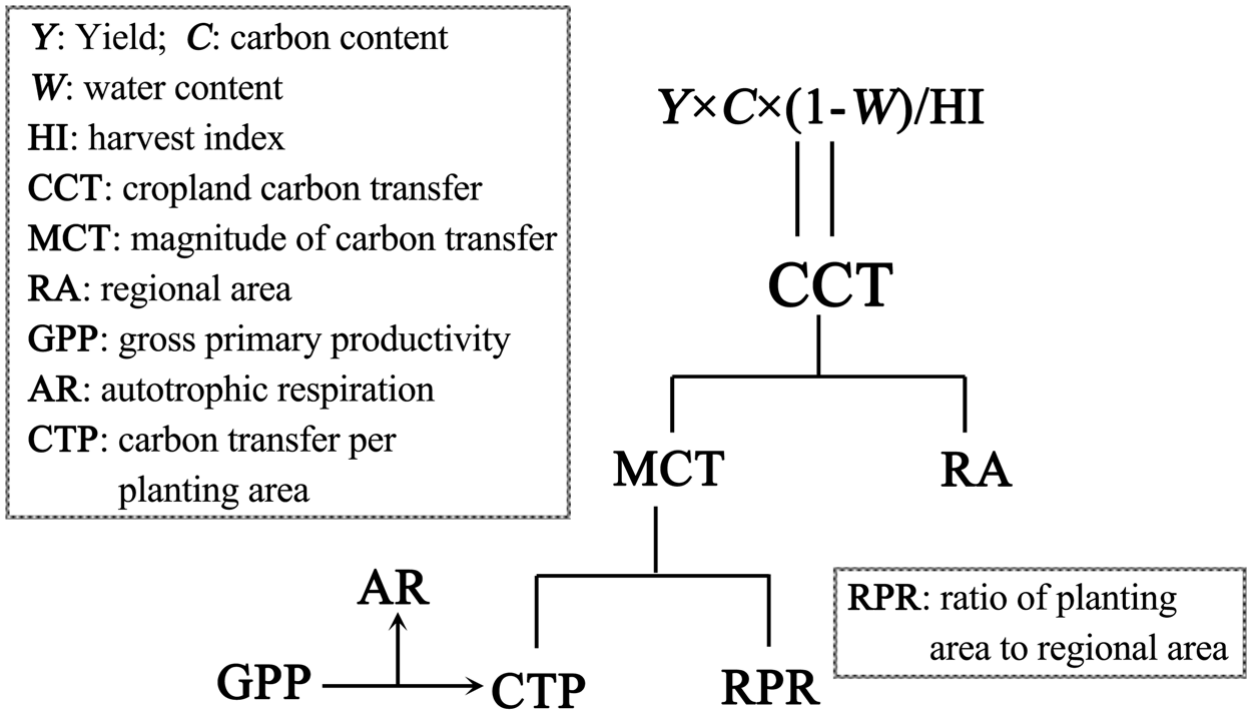
The components of cropland carbon transfer (CCT). The abbreviations of each item are listed in the box.

According to its definition, CCT is calculated from yield (*Y*), harvest index (HI), carbon content (*C*), and water content (*W*), where *Y* can be obtained from statistical yearbook and the remaining values are empirical parameters. In addition, CCT can be deemed as the product of the magnitude of carbon transfer per area (MCT, gC m^−2^ yr^−1^) and regional area (RA, m^2^), where RA can be obtained from available data. MCT, an important item in quantifying the regional carbon budget^14^, is thus calculated as the ratio of CCT to RA. Additionally, MCT is regarded as the product of carbon transfer per planting area (CTP, gC m^−2^ yr^−1^) and the ratio of planting area to regional area (RPR, %), where the planting area is obtained from statistical yearbook. Furthermore, CTP, the quantity of carbon contained in grains and straws but excluding residues, can also be deemed as gross primary productivity (GPP) minus autotrophic respiration (AR). Therefore, CCT can be separated into many components (Fig. 1). Analysing the roles of these components in the spatiotemporal variations of CCT can help understand the differences between its spatial and temporal variations.

Although many studies have intensively analysed the spatial and inter-annual variations of CCT using data from the China Statistical Yearbook^15–17^, they have paid little attention to the roles of CCT components in its spatial and temporal variations. Therefore, whether the drivers of CCT spatial variation differ from its temporal variation remains a puzzle, which inhibits the full understanding of its spatiotemporal variations and thus the difference between the spatial and temporal variations of ecological phenomena.

Given its high yield^18^, Liaoning province, located in the northeast of China, is one of the country’s most important producers of commodity grains thus guaranteeing national food security. Additionally, due to its particular climate, its carbon budget assessment contains substantial uncertainties^19^. Investigating the spatiotemporal variations of CCT in Liaoning province and the dominating components would provide data to support the carbon budget assessment, which would also help to illustrate whether the drivers of spatial variation differ from those of temporal variation.

Therefore, we analysed the spatiotemporal variations of CCT and their drivers in Liaoning province from 1992–2014, based on the Liaoning Statistical Yearbook. In addition, the factor decomposition model was employed to clarify the roles of CCT components in its spatiotemporal variations. The aims of our study were to clarify: (1) How CCT spatially and temporally varied in Liaoning province? (2) Which components dominated the spatial and temporal variations of CCT? (3) Were there differences in the drivers of spatial and temporal variations? Our results provide a data foundation to assess the regional carbon budget in Liaoning province and a reference for calculating CCT in other regions. They also provide evidence for understanding the difference between spatial and temporal variations.

## Results

### The spatial variations of CCT and its components

**Figure 2:**
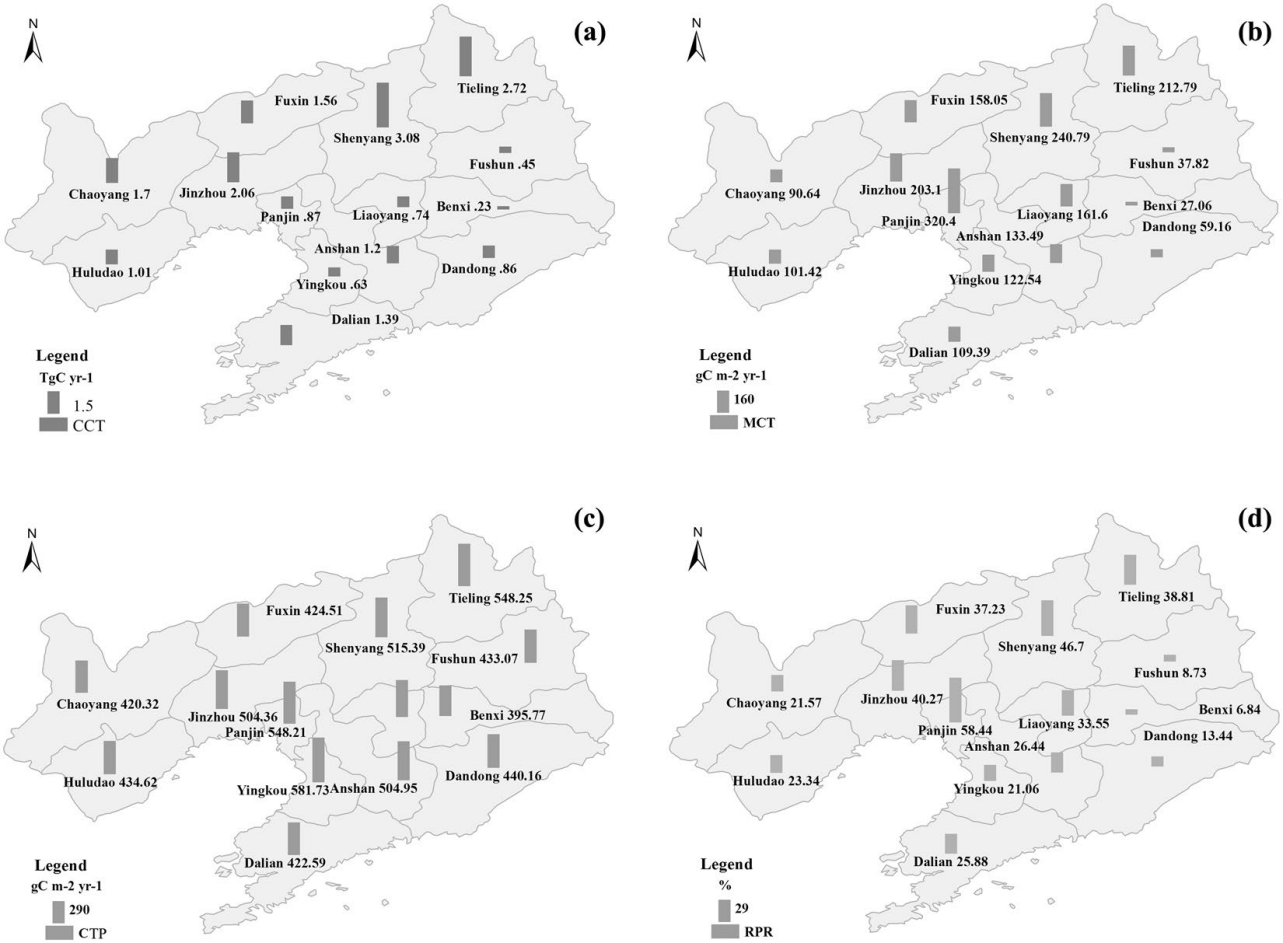
The spatial distributions of cropland carbon transfer (CCT, TgC yr^−1^, (**a**) and its components (**b–d**) in Liaoning province from 1992–2014. (**b–d**) Were the spatial distributions of the magnitude of carbon transfer per area (MCT, gC m^−2^ yr^−1^), carbon transfer per planting area (CTP, gC m^−2^ yr^−1^) and the ratio of planting area to regional area (RPR, %), respectively. The map was generated using ArcGIS 10.0 software.

From 1992–2014, the CCT of Liaoning province was 18.56 TgC yr^−1^ and spatially varied, exhibiting a decreasing trend from northwest to southeast (Fig. 2a). The northwest and west region, represented by Shenyang, Tieling, and Jinzhou, had the highest CCT, which exceeded 2 TgC yr^−1^, and each accounted for over 10% of Liaoning CCT. The CCT of the southeast and east region, represented by Fushun, Liaoyang, Benxi, Dandong, and Yingkou, were lower than 1 TgC yr^−1^, and each accounted for no more than 5% of the Liaoning CCT.

Factor decomposition model results suggest that the spatial variation of CCT was dominated by MCT, which accounted for 69.42% of CCT and RA accounted for the remained 30.58% of CCT spatial variation. MCT also varied spatially, with a decreasing trend from the centre to the border of Liaoning province (Fig. 2b). The central region, represented by Tieling, Shenyang, Jinzhou, and Panjin, had a higher MCT exceeding 200 gC m^−2^ yr^−1^. However, the border regions, such as Benxi, Dandong, Fushun, Dalian, Huludao, and Chaoyang, had a lower MCT, almost lower than 100 gC m^−2^ yr^−1^.

Factor decomposition model results further suggest that only 15.16% of the spatial variation of MCT was contributed by CTP, which, like MCT, exhibited an obvious spatial variation with a decreasing trend from the centre to the border. However, the relative variation of CTP between regions was smaller than that of MCT (Fig. 2c). The largest CTP, which was found at the central area of Liaoning province, such as Yingkou, Panjin, Tieling, and Shenyang, exceeded 500 gC m^−2^ yr^−1^, while the CTP of the border regions, such as Fushun, Dandong, Benxi, Chaoyang, and Huludao, also reached 400 gC m^−2^ yr^−1^.

In contrast with the small contribution of CTP, RPR, whose spatial variation followed a convex parabola from west to east (Fig. 2d), contributed 84.84% of the spatial variation of MCT. The highest RPR was found in the central areas of Liaoning province, such as Panjin, Shenyang, and Jinzhou, which exeeded 40%. The lowest RPR occurred in the eastern areas of Liaoning province, such as Fushun and Benxi, which were lower than 10%.

### The inter-annual variations of CCT and its components

**Figure 3:**
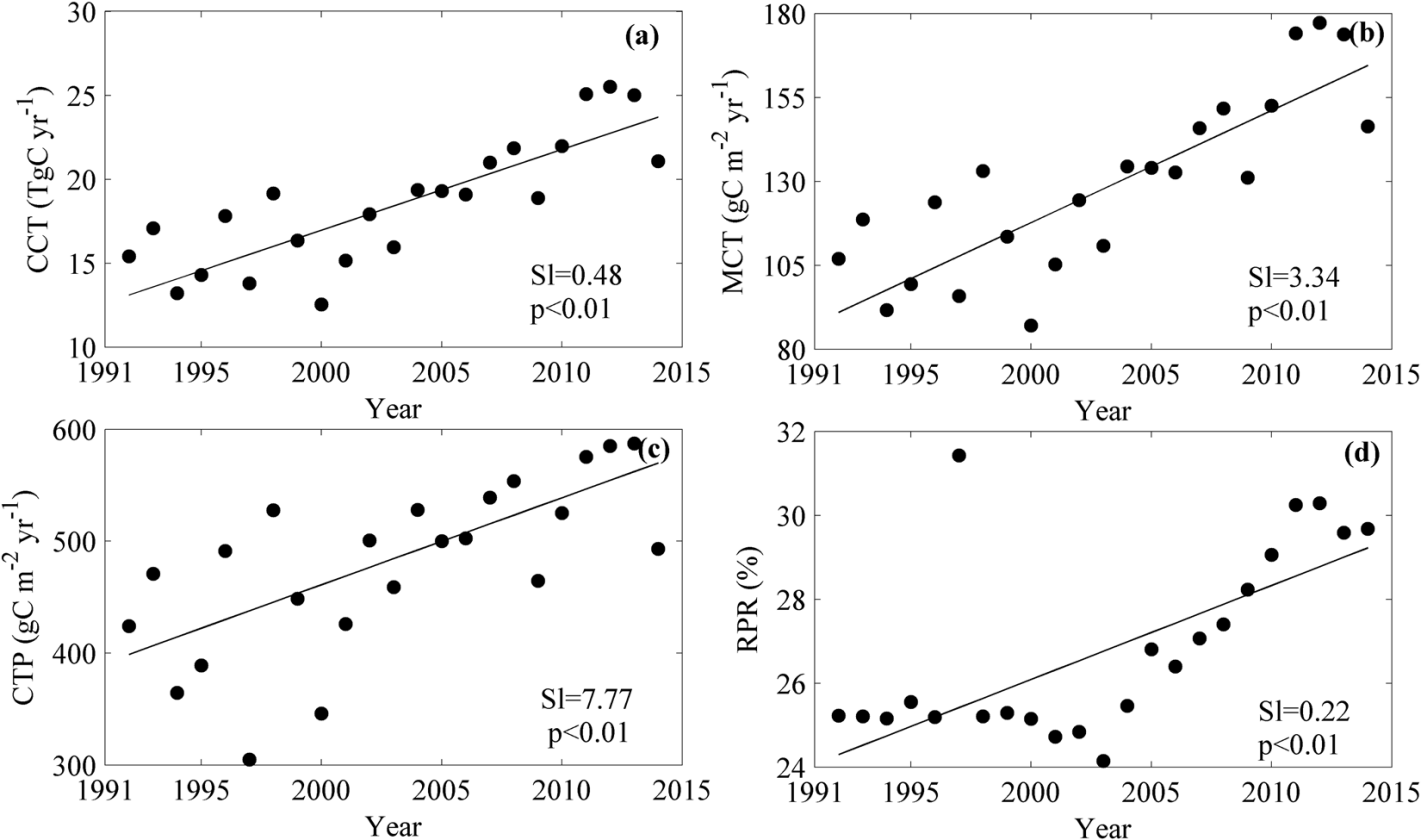
The inter-annual variations of cropland carbon transfer (CCT, Tgc yr^−1^, (**a**) and its components (**b–d**) in Liaoning province from 1992 to 2014. (**b–d**) were the inter-annual variations of the magnitude of carbon transfer per area (MCT, gC m^−2^ yr^−1^), carbon transfer per planting area (CTP, gC m^−2^ yr^−1^) and the ratio of planting area to regional area (RPR, %), respectively.

CCT showed a significant increasing trend among years (Fig. 3a). From 1992 to 2014, CCT increased at a rate of 0.48 TgC yr^−1^, accounting for 2.59% of annual CCT.

The given regional area (RA) varied little among years, and MCT dominated the pattern of CCT, exhibiting an increasing trend (Fig. 3b) at a rate of 3.34 gC m^−2^ yr^−1^.

Factor decomposition model results indicate that 62.64% of inter-annual MCT variation was contributed by CTP, which showed a significant increasing trend at a rate of 7.77 gC m^−2^ yr^−1^ from 1992 to 2014 (Fig. 3c). However, the contribution of RPR to inter-annual MCT variation was only 37.36%, though it also increased signficantly at a rate of 0.22% (Fig. 3d).

### Effects of factors on the spatiotemporal variations of CCT and its components

Climatic factors did not correlate significantly with the spatial variations of CCT and its components (Table 1). The correlation coefficients between the spatial variations of CCT, including its components, and climatic factors, such as sunshine duration (SD), annual mean air temperature (MAT), and annual mean precipitation (MAP), were lower than 0.4, indicating nonsignificant correlations at the level of 0.05. However, societal factors represented by fertilizer usage per area (FUA) correlated significantly with the spatial variations of CCT and its components. FUA had a positive correlation coefficient with CCT and its components, all exceeding 0.6, indicating a significant correlation at the level of 0.05.

**Table 1:** The correlations between cropland carbon transfer (CCT), including its components, and various factors.

CCT and its components	Factors	Spatial variation		Inter-annual variation	
		*r*	*p*	*r*	*p*
CCT	SD^2^	0.22	0.44	−0.17	0.43
	MAT^2^	−0.02	0.95	−0.23	0.29
	MAP^2^	−0.39	0.17	0.24	0.28
	**FUA** ^2^	**0**.**65** ^3^	**0**.**01**	**0**.**84**	**0**.**00**
MCT^1^	SD	0.33	0.24	−0.17	0.43
	MAT	0.28	0.33	−0.23	0.29
	MAP	−0.36	0.21	0.24	0.28
	**FUA**	**0**.**89**	**0**.**00**	**0**.**84**	**0**.**00**
CTP^1^	SD	0.18	0.55	−0.35	0.10
	MAT	0.23	0.44	−0.12	0.60
	MAP	−0.10	0.73	0.22	0.30
	**FUA**	**0**.**65**	**0**.**01**	**0**.**66**	**0**.**00**
RPR^1^	SD	0.38	0.18	0.28	0.19
	MAT	0.31	0.28	−0.26	0.22
	MAP	−0.42	0.13	0.09	0.67
	**FUA**	**0**.**90**	**0**.**00**	**0**.**76**	**0**.**00**

Note: ^1^MCT, CTP, and RPR were the abbreviations of the magnitude of carbon transfer per area, carbon transfer per planting area, and the ratio of planting area to regional area, respectively.

^2^SD, MAT, MAP, and FUA were the abbreviations of sunshine duration, annual mean air temperature, annual mean precipitation, and fertilizer usage per area, respectively.

^3^Significant correlations were indicated by bold numbers.

The effects of various factors on the inter-annual variations of CCT and its components were similar to their spatial variation (Table 1). With the changes of climatic factors, such as MAT, MAP, and SD, CCT and its components showed no significant variations, while the societal factors represented by FUA also increased CCT and its components among years.

## Discussion

**Figure 4:**
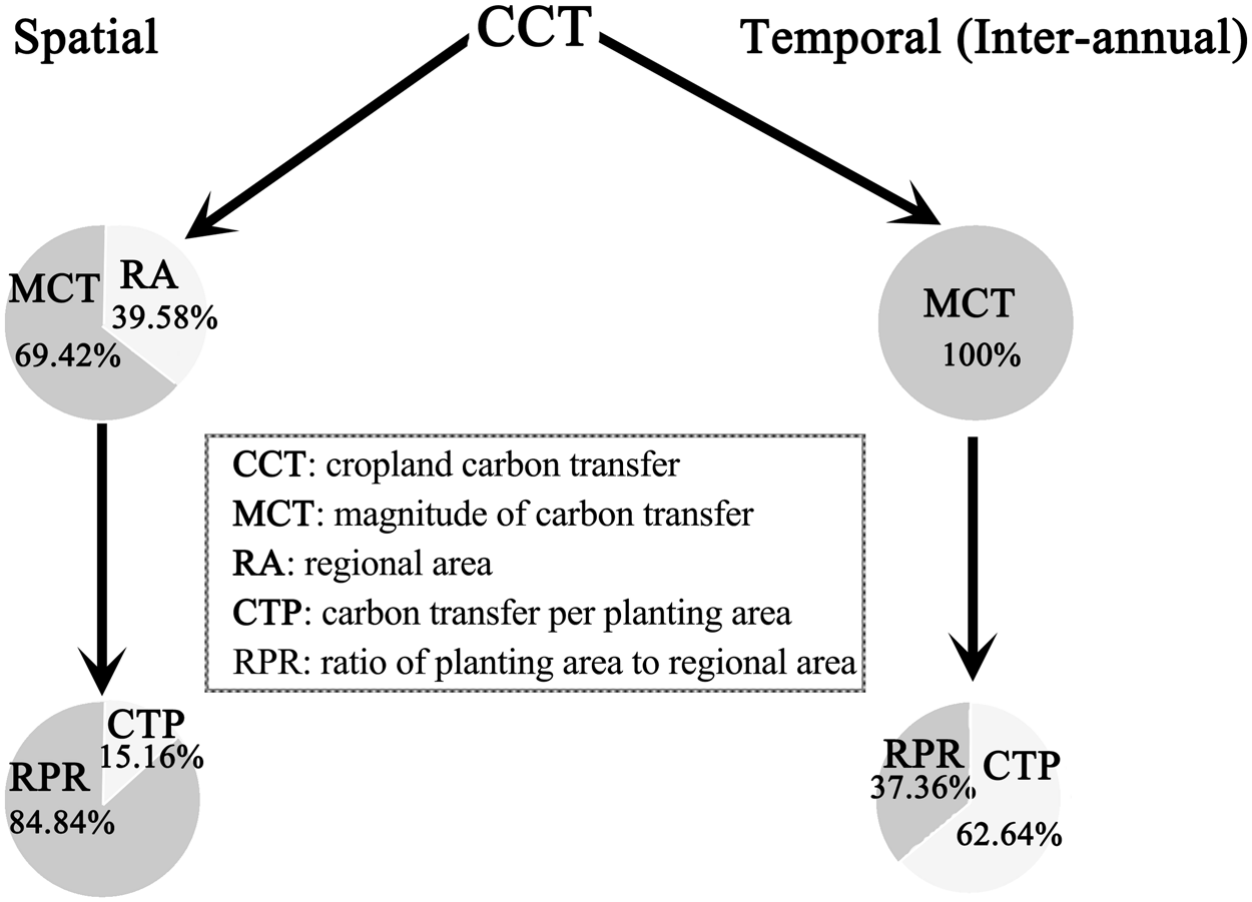
The roles of cropland carbon transfer (CCT) components in its spatial and temporal variations. The abbreviations of each item were listed in the box. Data were obtained from factor decomposition model.

In this study, we found CCT of Liaoning province decreased from northwest to southeast in spatial but obviously increased overall from 1992 to 2014. However, the components of CCT played different roles in the spatial and temporal variations of CCT (Fig. 4). RPR dominated the spatial variation of CCT through MCT, while CTP played a vital role in the inter-annual variation of CCT through MCT. This indicates that the roles of CCT components in its spatial variation differed from those in its inter-annual variation. Our findings were consistent with studies focusing on the spatiotemporal variations of gross primary productivity: the spatial variation of gross primary productivity was controlled by climate^5,6,20^ while its inter-annual variation was affected by the response of ecosystems to the varying climate^7,8,21^. This suggests that though substituting “temporal” with “spatial” has been an important practice in ecological studies, using conclusions from spatial analysis to infer temporal variation may overestimate the magnitude of temporal variation^4^. Therefore, we may need to separate temporal variation from spatial variation to investigate the spatiotemporal variations of ecological phenomena.

In addition, though climatic factors were not found to correlate significantly with the spatial variation of CCT, FUA did affect it, which may be ascribed to the following aspects. First, the spatial variation of CCT was dominated by that of RPR through its effect on MCT (Fig. 4). Second, climatic factors only significantly affected the spatial variation of GPP and AR and thus CTP, while CTP impacted a small portion of MCT and thus the spatial variation of CCT (Fig. 4). Thrid, FUA, the quantity of fertilizer used per area, can be deemed as the product of RPR and fertilizer usage per planting area, which made FUA correlate highly with RPR (Table 1) and thus CCT spatial variation through MCT (Fig. 4).

Though the effects of climatic factors and FUA on the inter-annual variation of CCT were similar to those on CCT spatial variation (Table 1), their effects may be ascribed to different aspects. First, the inter-annual variation of CCT was primarily affected by that of CTP through its effect on MCT (Fig. 4). Second, climatic factors may not be the direct factors driving the inter-annual variation of GPP^7,8,21^, which was the basis of CTP (Fig. 1). Third, the increasing FUA was accompanied by the increase in fertilizer usage per planting area, which made CCT increase from 1992 to 2014 through CTP, as fertilization may improve the crop yield.

## Conclusions

Based on data from the Liaoning Statistical Yearbook, we investigated the spatiotemporal variations of CCT and the factors that affected them. The results showed that the CCT of Liaoning province was 18.56 TgC yr^−1^ during 1992 to 2014, which showed a decreasing trend from northwest to southeast and increased at a rate of 0.48 TgC yr^−1^ from 1992 to 2014. The spatial variation of CCT was affected by FUA through its effect on RPR and thus MCT, while FUA affected the inter-annual variation of CCT through its effect on CTP and thus MCT. Therefore, the factors affecting the spatial variation of CCT differed from those that affected its temporal variation, indicating divergent drivers in the spatial and temporal variations of ecological phenomena.

## Methods

### CCT calculation

In this study, we calculated CCT as the product of crop yield (*Y*, g yr^−1^), harvest index (HI, g g^−1^), water content (*W*, gH_2_O g^−1^), and carbon content (*C*, gC g^−1^), since the statistical yearbook only reported the yield. Then, the CCT of Liaoning province was summed as CCT from all prefectural-level cities and all crops (Eq. (1)).

1 $${\rm{CCT}}=\sum _{i=1}^{14}\sum _{j=1}^{n}\{{Y}_{ij}\times (1-{W}_{ij})/{{\rm{HI}}}_{ij}\}\times {C}_{ij}$$

where *i* was the number of prefectural-level city and *j* was that of crops (Table 2), while *Y* of each prefectural-level city was obtained from the Liaoning Statistical Yearbook^22–44^.

**Table 2:** The values of harvest index (HI) and water content (*W*) of different crops.

Crops	Harvest Index (HI)	Water content (*W*)	References
Paddy	0.5	0.13	48–50
Maize	0.51	0.13	51–54
Wheat	0.46	0.13	16
Other cereal^1^	0.31	0.13	16
Millet	0.38	0.13	16
Sorghum^1^	0.31	0.13	16
Soybean	0.42	0.13	16
Yam^2^	0.64	0.133	16
Cotton	0.16	0.083	16
Peanut	0.50	0.09	16
Sesame	0.34	0.09	16
Sunflower	0.26	0.09	16
Other oil plants^3^	0.36	0.09	16
Sugarbeet	0.71	0.133	16
Tobacco	0.61	0.082	16
Vegetable	0.49	0.82	16

Note: ^1^HIs of Other cereal and Sorghum were calculated as the average HI of oat, triticale, and Rye in China^16^.

^2^HI of Yam was calculated as the average HI of potato, sweet potato, and cassava in China^16^.

^3^HI of other oil plants was calculated as the average HI of peanut, rape, sesame, and sunflower in China (Table 2).

HI, defined as the ratio of harvested grain to total dry matter^45^, differed among crops, which were listed in Table 2. The HI of paddy and maize were set as the average reported HI in Liaoning province, while for other crops, the average HI of that crop in China was selected, since little data was reported for Liaoning province.

*W* also differed among crops and were listed in Table 2 with values from previous studies^19,46^.

Though *C* somewhat differed among crops, the difference in *C* was small and little reported. We had no choice but to set *C* as 0.45 following the previous study^47^.

### Calculating CCT components

Given that CCT was the product of RA and MCT, MCT was calculated as:

2 $${\rm{MCT}}={\rm{CCT}}/{\rm{RA}}$$

where RA can be obtained from the raster calculator of ArcGIS.

In addition, MCT was regarded as the product of CTP and RPR, such that CTP can be calculated as:

3 $${\rm{CTP}}={\rm{MCT}}/{\rm{RPR}}$$

where RPR was calculated from the ratio of planting area, which can be found in the Liaoning Statistical Yearbook, to RA.

### Calculating the roles of CCT components in CCT spatiotemporal variations

In this study, we employed the factor decomposition model^55,56^ to distinguish the roles of CCT components in the spatiotemporal variations of CCT, which helped to illustrate the difference in the drivers of spatial and temporal varitions. The factor decomposition model separated specific variables, regarded as the multiplication of some parts, into its components using a logarithm way. Given that CCT was the product of RA and MCT, after taking the natural logarithm of the two sides, the relationship between CCT and its components was obtained as:

4 $${\rm{lnCCT}}={\rm{lnRA}}+{\rm{lnMCT}}$$

Therefore, the CCT of the benchmark region (CCT_0_) or any region (CCT_*i*_) can be expressed as:

5 $${{\rm{lnCCT}}}_{0}={{\rm{lnRA}}}_{0}+{{\rm{lnMCT}}}_{0}$$

6 $${{\rm{lnCCT}}}_{i}={{\rm{lnRA}}}_{i}+{{\rm{lnMCT}}}_{i}$$

After integrating Eqs (5) and (6), we can get the expression of CCT changes (∆lnCCT_*i*_) as:

7 $${{\rm{\Delta }}\mathrm{lnCCT}}_{i}={{\rm{lnCCT}}}_{i}-{{\rm{lnCCT}}}_{0}={{\rm{lnRA}}}_{i}-{{\rm{lnRA}}}_{0}+{{\rm{lnMCT}}}_{i}-{{\rm{lnMCT}}}_{0}$$

The amount of CCT variation from RA (∆CCT_RA_) and MCT (∆CCT_MCT_) can thus be expressed as:

8 $${{\rm{\Delta }}\mathrm{CCT}}_{{\rm{RA}}}=\sum _{i=1}^{n}({{\rm{CCT}}}_{i}-{{\rm{CCT}}}_{0})\times ({{\rm{LnRA}}}_{i}-{{\rm{LnRA}}}_{{\rm{0}}})/({{\rm{LnCCT}}}_{i}-{{\rm{LnCCT}}}_{{\rm{0}}})$$

9 $${{\rm{\Delta }}\mathrm{CCT}}_{{\rm{MCT}}}=\sum _{i=1}^{n}({{\rm{CCT}}}_{i}-{{\rm{CCT}}}_{0})\times ({{\rm{LnMCT}}}_{i}-{{\rm{LnMCT}}}_{{\rm{0}}})/({{\rm{LnCCT}}}_{i}-{{\rm{LnCCT}}}_{{\rm{0}}})$$

Therefore, the relative contribution of RA and MCT to CCT spatial variation can be expressed as ∆CCT_RA_/∆CCT and ∆CCT_MCT_/∆CCT, respectively. For simplicity, we selected the largest CCT_*i*_ for all prefectural-level cities as the benchmark value (CCT_0_).

The roles of CTP and RPR in the spatial and inter-annual variations of MCT were also investigated in the same way.

### Climatic and societal data

In this study, we selected the climatic data of each prefectural-level city (Table 3) from the Liaoning Statistical Yearbook^22–44^, including sunshine duration (SD), annual mean air temperature (MAT), and annual mean precipitation (MAP). In addition, we calculated the climatic data of Liaoning province based on climatic data for each prefectural-level city to illustrate the inter-annual variation of CCT.

**Table 3:** The mean climatic data of Liaoning province from 1992 to 2014.

Prefectural-level city	SD (hours)	MAT (°C)	MAP (mm)
Shenyang	2400.0	8.43	684.3
Dalian	2625.6	11.41	619.5
Anshan	2560.4	10.45	714.3
Fushun	2506.7	7.10	775.7
Benxi	2548.5	8.27	798.8
Dandong	2387.7	9.33	1000.0
Jinzhou	2648.4	10.23	557.1
Yingkou	2648.2	9.96	627.3
Fuxin	2638.8	8.23	475.2
Liaoyang	2306.9	9.34	687.5
Panjin	2602.8	9.35	596.2
Tieling	2623.0	8.37	633.9
Chaoyang	2606.8	9.70	478.1
Huludao	2573.5	10.03	564.9

Note: SD, MAT, and MAP were the abbreviations of sunshine duration, annual mean air temperature, and annual mean precipitation, respectively.

Furthermore, given that fertilization promoted crop yield and thus CCT, we selected fertilizer usage per area (FUA) as a societal factor affecting the spatiotemporal variations of CCT. FUA was calculated using the regional area and the quantity of fertilizer used in each prefectural-level city, as reported in the Liaoning Statistical Yearbook.

### Statistical analysis

In this study, we created the spatial distributions of CCT, MCT, RPR, and CTP with ArcGIS 10.0. The trends of CCT, MCT, RPR and CTP were determined by Mann-Kendall trend analysis using MATLAB 2014 (MathWorks Inc., Natick, MA, USA). The correlation between CCT, including its components, and various factors, including climatic and societal variables, were investigated with correlation analysis using MATLAB 2014 at the significance level of 0.05.

### Data availability statement

The datasets analysed during the current study are calculated based on the methods described in this study and the original data of the statistical yearbook, which can be found at www.cnki.net. All datasets generated during the current study are available from the corresponding author on reasonable request.
